# Evaluating the contribution of historical and contemporary temperature to the oospore production of self‐fertile *Phytophthora infestans*

**DOI:** 10.1111/eva.13643

**Published:** 2024-01-29

**Authors:** Abdul Waheed, Lin‐Lin Shen, Oswald Nkurikiyimfura, Han‐Mei Fang, Yan‐Ping Wang, Björn Andersson, Jiasui Zhan, Li‐Na Yang

**Affiliations:** ^1^ Fujian Key Laboratory on Conservation and Sustainable Utilization of Marine Biodiversity, Fuzhou Institute of Oceanography Minjiang University Fuzhou China; ^2^ Institute of Plant Pathology Fujian Agriculture and Forestry University Fuzhou China; ^3^ College of Chemistry and Life Sciences Chengdu Normal University Chengdu China; ^4^ Department of Forest Mycology and Plant Pathology Swedish University of Agricultural Sciences Uppsala Sweden

**Keywords:** adaptation, agriculture, climate change, ecological genetics, evolution of sex, microbial biology

## Abstract

Reproductive systems play an important role in the ecological function of species, but little is known about how climate change, such as global warming, may affect the reproductive systems of microbes. In this study, 116 *Phytophthora infestans* isolates sampled from five different altitudes along a mountain were evaluated under five temperature regimes to determine the effects of historical and experimental temperature on the reproductive system of the pathogen. Both altitude, a proxy for historical pathogen adaptation to temperature, and temperature used in the experiment affected the sexual reproduction of the pathogen, with experimental temperature, that is, contemporary temperature, playing a role several times more important than historical temperature. Furthermore, the potential of sexual reproduction, measured by the number of oospores quantified, increased with the temperature breadth (i.e., difference between the highest and lowest temperature at which sexual reproduction takes place) of the pathogen and reached the maximum at the experimental temperature of 21°C, which is higher than the annual average temperature in many potato‐producing areas. The results suggest that rising air temperature associated with global warming may increase the potential of sexual reproduction in *P. infestans*. Given the importance of sexuality in pathogenicity and ecological adaptation of pathogens, these results suggest that global warming may increase the threat of *P. infestans* to agricultural production and other ecological services and highlight that new epidemiological strategies may need to be implemented for future food security and ecological resilience.

## 1 INTRODUCTION

The global climate is undergoing unprecedented changes. Air temperature is projected to increase by several degrees by the end of the century if the current rate of change continues (Raftery et al., 2017). UV radiation has also increased continuously in recent decades (Neale et al., 2021). There is a widespread concern that these changes, together with their associated secondary and tertiary climatic and environmental events such as increasing frequency and duration of droughts or floods, rising sea levels and elevated soil salinity, etc., can have devastating effects on social and natural sustainability including food production, human health, economic development and landscape aesthetics by affecting the structure, function, biodiversity and resilience of ecosystems (Ortiz et al., 2021).

The extent of the impacts of climate change on social and environmental sustainability is mainly determined by the genetic variation in the species that make up the ecosystem. Adaptation requires species to be able to rapidly adjust phenotypic traits to meet environmental changes. Fisher's fundamental theorem of natural selection for adaptation states that the ability and rate of adaptation to stress induced by changing environments depend on genetic variations in ecological and biological properties relevant to species fitness (Fisher, 1930). Higher population variation in such traits increases the presence of phenotypes favoured by different environments and therefore facilitates adaptation. This genetic variation can be generated through changes in genome composition and expression. Change in genome composition, usually measured by heritability, can result in permanent adaptation to a specific environment (Lalejini et al., 2021), while change in gene expression, reflected by phenotypic plasticity, is a phenomenon in which a genotype can generate a series of phenotypes in responding to transient environmental fluctuations. The relative importance of the two genetic events in species adaptation is trait and phase dependent (Alster et al., 2021). Phenotypic plasticity is particularly important in (i) species adaptation to rapid environmental change such as air temperature and UV radiation (Wu et al., 2019; Yang et al., 2016) due to its immediate response and (ii) the early phase of evolutionary adaptation, which would be reinforced later by genomic changes (Ho & Zhang, 2018).

As a driver of evolution, the reproductive system plays an important role in the adaptation of species to climate change. It achieves this by regulating genetic variation and generating stress tolerance propagules. Asexually reproducing species generally have less genetic variation than their sexual counterparts. Sexual reproduction increases the genotypic variation of species by generating new genotypes through intergenic recombination (Hall et al., 2010) or even new alleles through intragenic recombination (Shen et al., 2021). It also prevents the loss of genetic variation caused by hitchhiker selection (Kim & Stephan, 2002) and the Muller‐Ratchet effect, that is, the accumulation of deleterious mutations that could lead to population collapse (Higgins & Lynch, 2001). The theory of evolution assumes that sexual reproduction is derived from asexual reproduction and that almost all multicellular organisms and various unicellular organisms employ sexual reproduction as a sole or complementary reproductive strategy despite its fitness cost (Judelson, 2009). In many plant and eukaryotic microbes, sexual propagules such as seeds and oospores are more stress tolerant compared to vegetative propagules. They can remain viable in soils or other environments for many years (Barwell et al., 2021) and disperse over long distances, for instance, by trade and air circulation, to colonize new territories.

Reproductive strategy is a key life‐history trait with a rich genetic and ecological context. In genetics, reproductive strategy involves a cascade of biochemical signalling pathways that mediate hormone production, energy homeostatic and the development of certain reproductive structures (Niu et al., 2018; Tomura et al., 2017). At the ecological level, reproductive strategy is regulated by demography, nutritional availability and climatic factors (Corredor‐Moreno & Saunders, 2020). In species capable of both sexual and asexual reproduction, the interaction between the genetic and environmental factors determines the relative abundance of the two reproductive strategies and may vary temporally and spatially among populations (Newman & Derbyshire, 2020).

Research in reproductive strategy has mainly focused on biochemical and molecular aspects such as hormones and genes involved (Niu et al., 2018; Tomura et al., 2017). Knowledge of how environmental factors such as air temperature affect the reproductive strategy is generally limited but important to project the impact of climate changes such as global warming on social and ecological sustainability. It has been documented that temperature can exert a vital influence on the reproductive strategy of species (Maynard et al., 2019; Zhan & McDonald, 2011). Interaction between temperature and reproductive strategy was also found in many other species (Shaffer et al., 2020).

Eukaryotic microbes provide excellent models to study reproductive strategies regulated by climate change and its social and ecological impacts. Many eukaryotic microbes evolve a mixed mode of reproduction, in which sexual reproduction produces new adaptive variants, and co‐adapted traits are retained through asexual reproduction. This allows microbes to replicate rapidly and generate very large population sizes that can be instantly exposed to defined selective conditions, fulfilling the key requirements for experimentally assessing the role of climate change in the evolution of biological and ecological traits. Infectious agricultural pathogens represent an additional advance for the study as they are biologically and genetically well characterized and have a mature molecular and genomic toolbox that allows precise biological and ecological analysis.

In this study, we used *Phytophthora infestans*, one of the most destructive plant pathogens worldwide (Fry, 2008), to investigate the influence of historical and contemporary (experimental) temperature on oospore production and infer the impact of global warming on social and ecological sustainability. This pathogen caused the great Irish famine during the 1840s and is still responsible for billions of US dollars in economic loss each year (Dong & Zhou, 2022). *Phytophthora infestans* is a heterothallic oomycete with facultative reproduction in which sexually formed oospores are produced by the fusion of two opposite mating types designated as A1 and A2. Self‐fertile individuals in which sexual reproduction can be completed without the involvement of a mating partner have also been documented in the pathogen and dominate in some parts of the world such as in Yunnan, China (Zhu et al., 2016). For some oomycete species such as *P. capsica* (Hurtado‐Gonzales & Lamour, 2009), sexual spores may be produced from apomixis, although this phenomenon has not yet been documented in *P. infestans*. In addition to generating new genotypes and alleles, oospores are stress tolerant and can remain viable in soil or plant debris for several years (Turkensteen et al., 2000) to serve as primary inoculum (Grunwald & Flier, 2005). Previous research shows that β‐glucanases, NF‐Y transcription factor, histone and many other genes are involved in α‐hormones biosynthesis, cell wall degradation and other processes during oospore production and germination (Niu et al., 2018; Tomura et al., 2017). Temperature and other environmental factors are also associated with oospore production of the pathogen (Barwell et al., 2021; Cohen et al., 1997; Turkensteen et al., 2000).

The specific objectives of our study were to: (1) determine the altitudinal distribution of oospore production in *P. infestans*; (2) evaluate the contribution of contemporary and historical temperature to *P. infestans* oospore production; (3) construct the thermal profile of oospore production in *P. infestans*; and (4) infer the influence of global warming on future reproductive strategy of *P. infestans*. Results from this study can provide insights into the impacts of climate change on ecosystem and species adaptation mechanisms. This knowledge is important to support the development of climate mitigation strategies to support societal and ecological sustainability such as future food security and biodiversity.

## 2 MATERIALS AND METHODS

### 2.1 *Phytophthora infestans* collection

Potato leaves infected by *P. infestans* were sampled from five altitudes along the Dongshan Mountain located in Xuanwei, Yunnan, during the mid‐2016 late blight epidemic season. The five locations, ranging from 1976 to 2591 m in altitude, were designated as A to E from lower to higher altitudes respectively (Table 1). For all collections, infected leaves were sampled randomly from potato plants at a distance of 1–2 m and transferred to the laboratory within 24 h for pathogen isolation. To isolate the pathogen, infected leaves were first washed under running water for 60 s, then with sterilized distilled water for 30 s and placed abaxial side up on 1.0% water agar for 20–30 h. A single piece of mycelium was aseptically removed from a sporulating lesion using a seed needle, transferred to rye B agar supplemented with ampicillin (100 μg/mL) and rifampicin (50 μg/mL) and incubated at 19°C for 7 days in dark to develop a colony. Each isolation was purified by sequential transfers of a single sporangium to a fresh rye B plate supplemented with ampicillin (100 μg/mL) and rifampicin (50 μg/mL). A total of 354 isolates were secured from the collection, with 59 to 87 isolates originating from each of the five locations. Detailed information on sample collection, pathogen isolation and purification can be found in previous publication (Yang et al., 2016).

**TABLE 1:** Least significant difference test for differences in oospore production on the 15th day after inoculation and estimated maximum numbers of oospore (MNO) production of the *Phytophthora infestans* sampled from five altitudes along the Dongshan Mountain in Xuanwei, Yunnan.

Sites	Sample size	Altitude (m)	13°C	16°C	19°C	22°C	25°C	MNO
A	25	1975	0.44^B^	15.84^A^	44.16^A^	172.03^B^	0.32^A^	86.83^B^
B	27	2124	0.88^A^	13.35^A^	32.69^A^	268.35^A^	0.22^A^	119.78^A^
C	25	2471	0.84^A^	6.03^B^	42.07^A^	158.04^B^	0.08^A^	80.52^B^
D	17	2591	0.29^B^	4.88^BC^	39.08^A^	166.29^B^	0.29^A^	82.16^B^
E	22	2677	0.29^B^	2.47^C^	42.29^A^	278.64^A^	0.23^A^	124.97^A^
Average	23.3	2368	0.55	8.51	40.06	208.67	0.23	98.85

*Note*: Values followed by different letters in a column are significantly different from each other at *p* = 0.05.

### 2.2 Molecular genotyping and mating type assays of *Phytophthora infestans*

A 100 mg weight of mycelia was collected from 15 days of culturing the pathogen on rye B agar plates at 19°C under the darkness and shifted into 2 mL sterile centrifuge tubes and lyophilized with a vacuum freeze dryer (Alpha1‐2, Christ). A mixer mill (MM400, Retsch) was used to ground the lyophilized mycelia. Plant gDNA Miniprep Kit (GD 2611, Biomiga, China) was used to extract the DNA according to the company's instructions. The genomic DNA was preserved in 200 μL pure water and kept at −20°C for future experimental processes. Eight pairs of SSR primers (Markers) (Pi02, Pi04, Pi4B, PiG11, Pi16, Pi33, Pi56 and Pi89) were used to amplify the genomic DNA of each isolate as reported (Knapova & Gisi, 2002), and DNA amplifications for SSR genotyping were performed by following the protocols of our previous publication (Qin et al., 2016).

The mating type was determined by growing each isolate separately and with reference isolates of A1 and A2 mating types on rye agar plates at 19°C under dark conditions. After 2 weeks of inoculation, the mycelium was picked either from the junctions of two colonies between the test and reference isolates or from the edges of the colonies for isolates grown alone to observe the oospore generations. Isolates are assigned to the opposite mating type from the one they form oospores with, that is, unknown isolates of mating type A1 form oospores with A2 reference isolate, but not with A1 reference isolate, and vice versa for unknown isolates of mating type A2 (Cooke et al., 2003). Isolates are considered self‐fertile when they can produce oospores without the involvement of a mating partner.

### 2.3 Measuring oospore production in *Phytophthora infestans*

All isolates from these Yunnan collections were tested to be self‐fertile although A1 and A2 mating types have been detected in other parts of China (Zhu et al., 2016). Among them, 116 isolates with different genotypes were selected for the oospore production assay, with 17–27 isolates from each of the five populations (Table 1). These self‐fertile *P. infestans* isolates were revived from long‐term storage on rye B agar at 19°C for 10 days. Mycelia plugs (0.3 cm in diameter) were taken from the margin of each revived colony and inoculated onto new rye B plates in a 9 cm Petri dish. The inoculated plates were exposed to one of five experimental temperatures (13, 15, 19, 22 and 25°C) in growth chambers and were laid out in a completely randomized design using three replicates as recommended by previous studies (Yang et al., 2016; Zhan & McDonald, 2011). After 15 days of culture, a plug of mycelium with a diameter of 0.6 cm was taken from the edge of the colony. Mycelia were carefully removed from the plug with a sterile toothpick, transferred to glass slide containing a drop of sterilized distilled water and then covered with coverslip. Three microscopic slides were prepared from each isolate and the number of oospores in each slide was counted manually using a light microscope (NIKON Ni‐U) at 10× magnification. Overall, 1740 (116 isolates × 3 replicates × 5 temperatures) experimental units were included to estimate the number of oospore production. To minimize errors, each experimental step within a particular temperature scheme, such as inoculation and spore counting of the isolates, was performed by the same person on the same day.

### 2.4 Data analysis

The thermal reaction norm of oospore production in each isolate was fitted to a second‐order polynomial distribution using the values generated in the five experimental temperatures. The resulting norms were used to estimate minimum temperature (OT_min_), optimum temperature (OT_opt_), maximum temperature (OT_max_), temperature breadth (OT_b_) and maximum oospore production (MNO) as described previously (Wu et al., 2022; Yang et al., 2016). Briefly, OT_max_ and OT_min_ of oospore production were estimated from the thermal reaction norm by setting the quadratic equation to zero and then solving it. OT_opt_ was estimated by taking the first derivative of the quadratic equation, setting it to zero and then solving it, which was then used to project the maximum potential of sexuality, that is, MNO, of the isolates and OT_b_, which was determined by taking the difference between OT_max_ and OT_min_. Variances in oospore production were analysed and partitioned into sources attributable to ‘isolate’ (I, random effect), ‘altitude’ (P, random effect) and ‘experimental temperature’ (T, random effect) using SAS GLM and VARCOMP programs (SAS 9.3 Institute) according to the general linear model:

(1) $$Y_{\text{ript}}=M+I\left(P\right)+T+P+I\left(P\right)\times T+P\times T+E_{\text{ript}}$$

Where Y_ript_ is the mean oospore production of replicate *r* for Isolate *i* from Altitude *p* at Experimental Temperature *t*; M is the overall mean; T is the experimental temperature; and E_ript_ is the variance among replicates. The terms P, I(P), I(P) × T and P × T refer to genetic variance among altitudes, genetic variance within altitudes, variance due to the interaction between isolate and experimental temperature and different responses of altitudinal populations to changing experimental temperature respectively. GLM also was used to analyse variance in OT_min_, OT_opt_, OT_max_, OT_b_ and MNO.

Population differentiation (*Q*_ST_) in oospore production between altitudinal populations was estimated by partitioning genetic variation attributable to population variation in response to experimental temperatures (Zhan et al., 2005).

(2) $$Q_{\mathrm{ST}}=\frac{\delta_{\mathrm{AP}}^{2}+\left(\delta_{P\cdot E}^{2}\right)/n}{\delta_{\mathrm{AP}}^{2}+\left(\delta_{P\cdot E}^{2}\right)/n+2\delta_{\mathrm{WP}}^{2}}$$

Where *δ* ^2^_AP_, *δ* ^2^_P.E_ and *δ* ^2^_wp_ are the additive genetic variation attributable to among‐altitude difference, interaction between altitudinal population and experimental temperature, difference within altitudinal population variation, respectively, and *n* is the number of experimental temperatures. Both pairwise and overall *Q*_ST_ were estimated for the altitudinal populations. Heritability of oospore production in an altitudinal population was calculated by dividing genetic variance within the population, that is, I(p) in Equation 1 above, with the total phenotypic variance. Phenotypic plasticity of oospore production in an altitudinal population was estimated by dividing the variance of isolate–temperature interaction in the population by total phenotypic variance (Tonsor et al., 2013). Population differentiation (*F*_ST_) in the neutral genome was estimated from the eight SSR marker loci used for genotype detection by the fixation index (Meirmans & Hedrick, 2011) using POPGENE 1.32. Standard deviation of the overall *Q*_ST_ was constructed by 100 resamples with replacement of original data as described previously (Zhan & McDonald, 2011) and was used to determine the evolutionary history of oospore production by a *t*‐test between the population genetic differentiations in SSR loci and oospore production. Contributions of historical temperature to oospore production and development of thermal biology in *P. infestans* were evaluated by the least significant difference (LSD) test and association analysis between oospore production and altitude (Ott & Longnecker, 2015).

## 3 RESULTS

### 3.1 Phenotypic variation in oospore production of *Phytophthora infestans*

**FIGURE 1:**
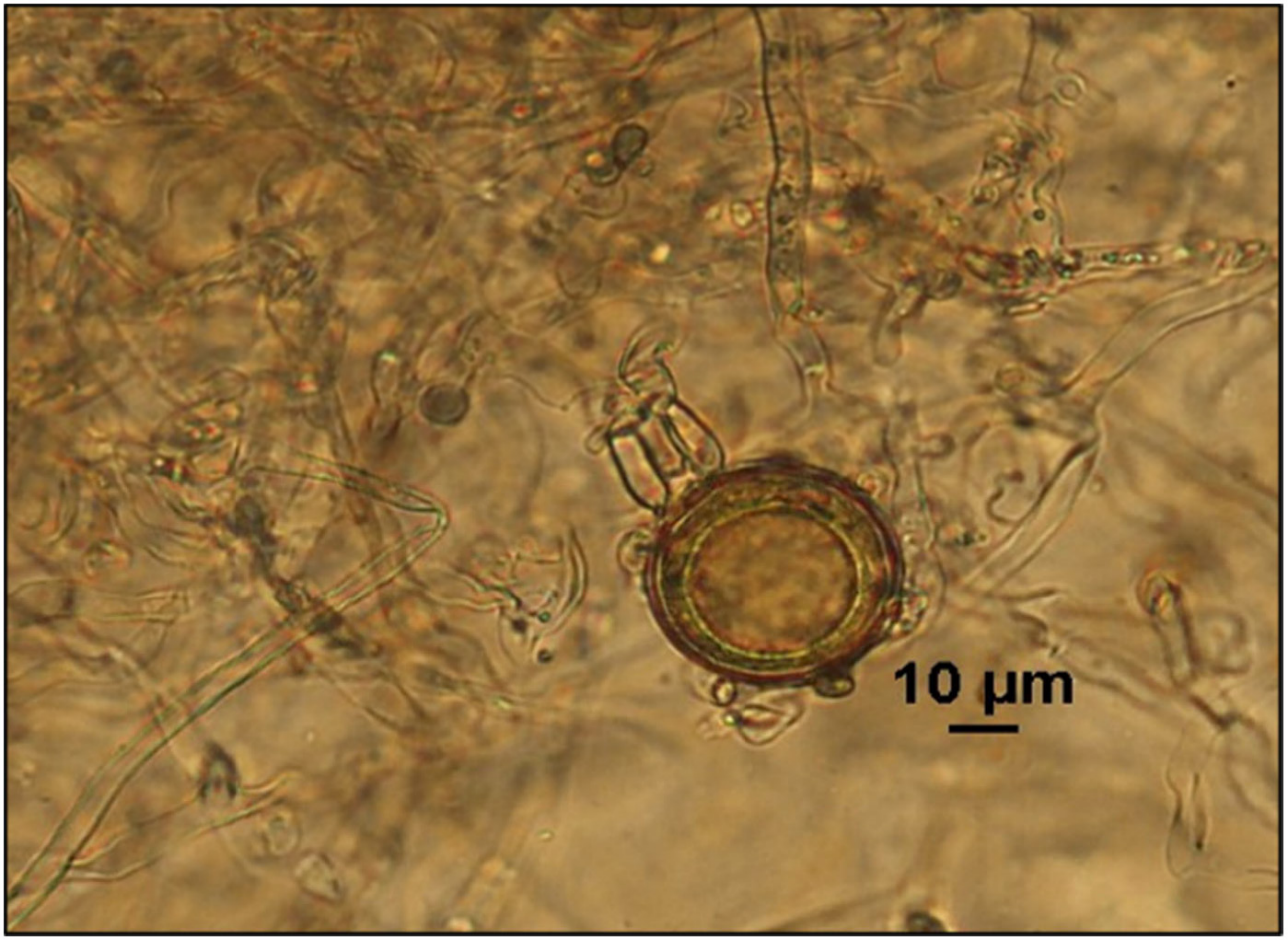
The oospore morphology of *Phytophthora infestans*. Bars = 10 μm.

A total of 116 *P. infestans* isolates sampled from the five altitudinal sites along a mountain were tested for oospore production under five experimental temperatures. Oospores (Figure 1) were detected in all isolates after 15 days of in vitro culture, yielding 1736 data points of 1740 (116 isolates × 5 temperatures × 3 replicates) expected (Table S1). Contamination during pathogen culture resulted in the loss of the four data points. Analysis of variance by the general linear model indicated that ‘isolate’ (i.e. genotype), ‘altitude’ (i.e. historical temperature) and ‘experimental temperature’ all contributed significantly (*p* < 0.0001) to the oospore production of *P. infestans* (Table S2). Among the three primary factors, experimental temperature had the greatest effect on oospore production, followed by altitude (historical temperature) and isolate (genotypes). The pathogen isolates from different altitudes responded differently to experimental temperatures in oospore production. Altitude–temperature was more important than the isolate–temperature interaction (*p* < 0.0001) in regulating oospore production of *P. infestans* (Table S2).

### 3.2 Thermal reaction norm of oospore production in *Phytophthora infestans*

**FIGURE 2:**
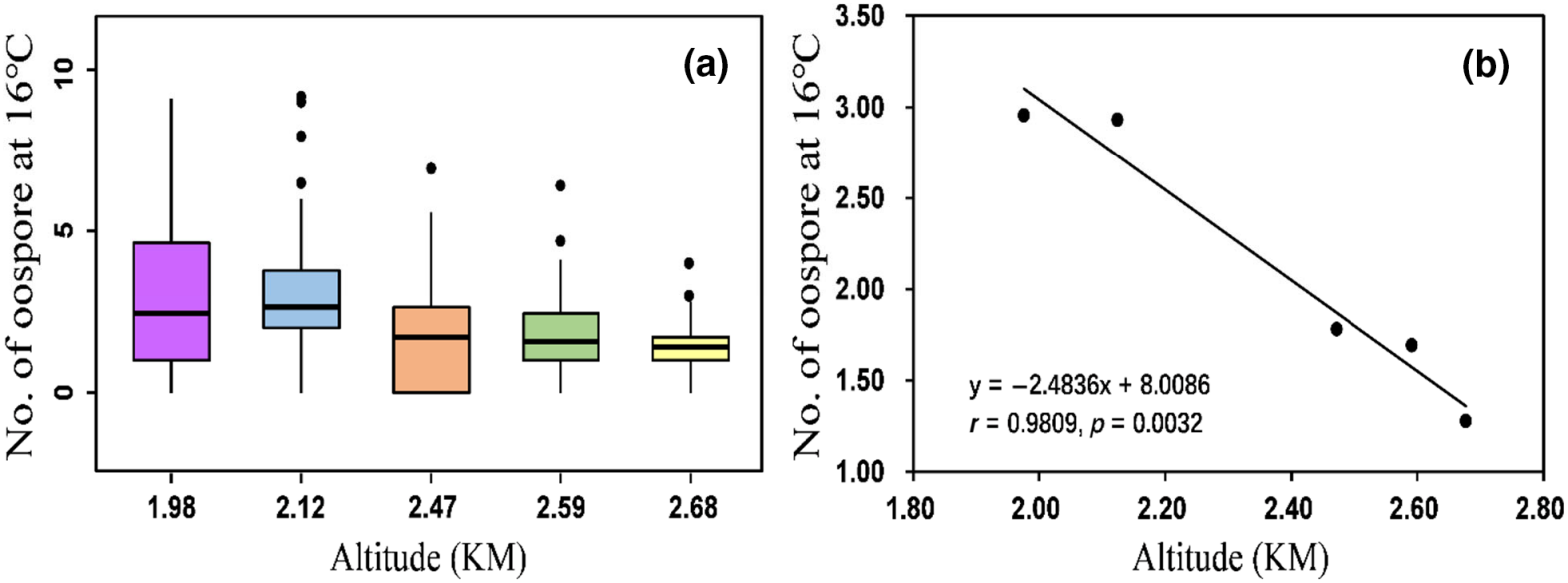
Linear association between oospore production and altitudinal origin of *Phytophthora infestans* at the experimental temperature of 16°C.

Although oospores were detected in all five temperature regimes used in the experiment, they were mainly produced at temperatures between 16 and 22°C (Table 1). Oospore production gradually increased from 13°C, reached a peak at 22°C and then sharply decreased as the experimental temperature further increased to 25°C. When the experimental temperature was 16°C, there was an altitudinal pattern of oospore production and the production of oospores decreased with the increase in altitude (Table 1, Figure 2). No such altitudinal variation was detected in the other two experimental temperatures with meaningful oospore production.

**FIGURE 3:**
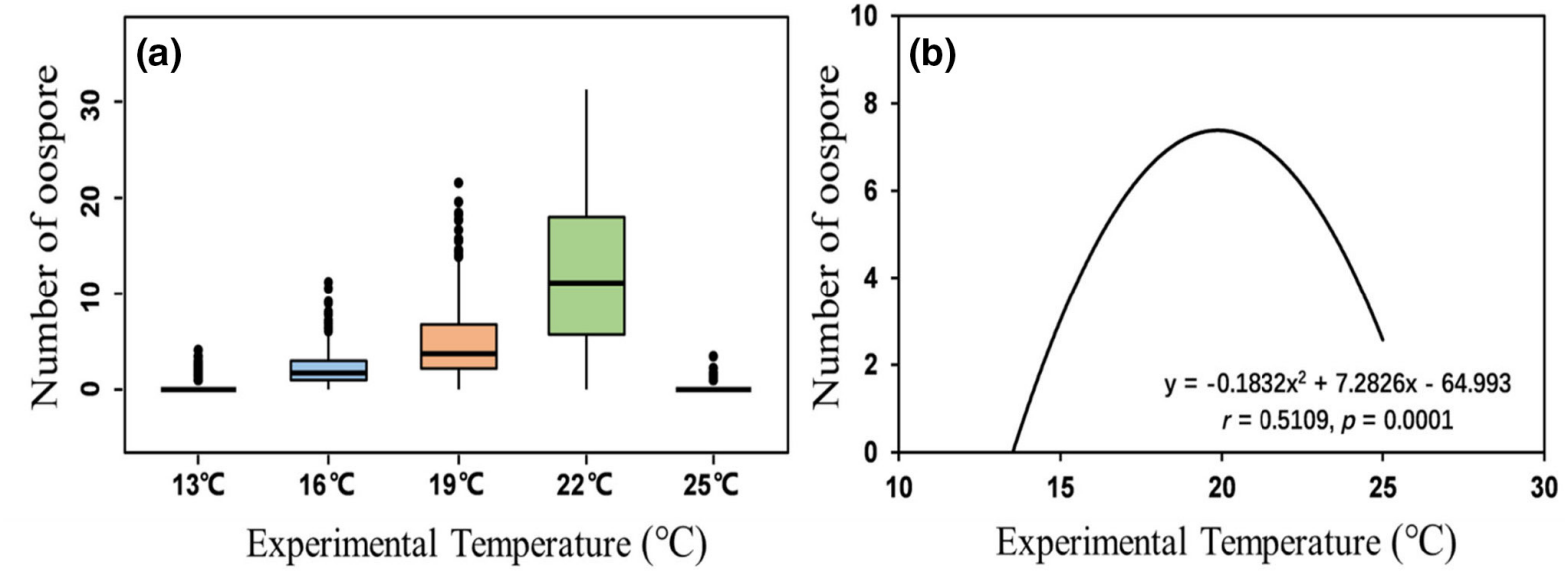
The thermal reaction norm of oospore production in the *Phytophthora infestans* isolates sampled along an altitudinal gradient of the Dongshan Mountain located in Xuanwei, Yunnan, China.

**FIGURE 4:**
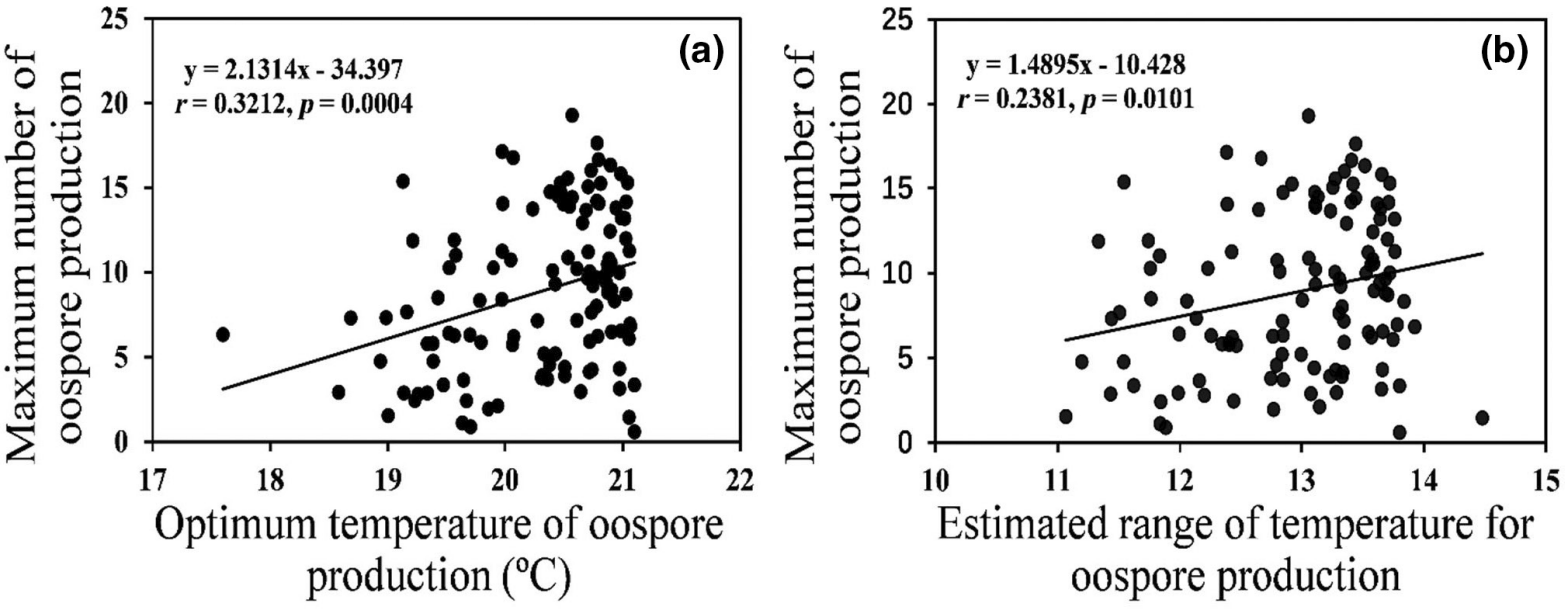
Associations of the estimated maximum oospore production with thermal biology of *Phytophthora infestans*: (a) estimated optimum temperature and (b) estimated temperature breadth.

Thermal reaction norm of oospore production fitted well to a quadratic polynomial model (Y = −0.1832x^2^ + 7.2826x − 64.993, *p* = 0.0001 Figure 3). Further statistical analysis showed that these thermal parameters of oospore production in the pathogen were influenced significantly by ‘altitude’, but only marginally by ‘isolate’ (Table S3). On average, OT_max_, OT_opt_, OT_min_ and OT_b_ in the pathogen were 26.76, 20.29, 13.82 and 12.94°C, respectively, although substantial differences existed among isolates and populations from different altitudes (Table 2). OT_max_ in the *P. infestans* isolates was positively associated with their estimated OT_opt_ (*r* = 0.3212, *p* = 0.0004, Figure 4a) and OT_b_ (*r* = 0.2381, *p* = 0.0101, Figure 4b).

**TABLE 2:** Least significant difference test for differences in the estimated maximum temperature (OT_max_), optimum temperature (OT_opt_), minimum (OT_min_) temperature and temperature breadth (OT_max_ − OT_min_) of oospore production in the five *Phytophthora infestans* populations sampled from five altitudinal sites (ranged from lowest altitude at the site A to highest altitude at the site E) along the Dongshan Mountain located in Xuanwei, Yunnan, China.

Sites	OT_opt_ (°C)	OT_max_ (°C)	OT_min_ (°C)	OT_max_ − OT_min_
A	20.24^B^	26.77^B^	13.70^D^	13.07^A^
B	20.45^A^	27.05^A^	13.85^B^	13.20^A^
C	20.06^C^	26.34^C^	13.77^BC^	12.57^C^
D	20.20^B^	26.60^B^	13.80^BC^	12.80^B^
E	20.51^A^	27.04^A^	13.99^A^	13.05^A^
Average	20.29	26.76	13.82	12.94

*Note*: Values followed by different letters in a column are significantly different from each other at *p* = 0.05.

### 3.3 Quantitative genetic analysis of oospore production in *Phytophthora infestans*

Genetic variance (heritability), which was estimated by partitioning the phenotypic variance, accounted for 0.007–0.062 of the phenotypic variation in oospore production of the pathogen from an altitude with a mean of 0.029 while the variance of the isolate–temperature interaction (plasticity) accounted for 0.702–0.831 of the phenotypic variation with a mean of 0.736 (Table 3). Plasticity was 12‐ to 99‐folds (average 25) higher than heritability (Table 3).

**TABLE 3:** Heritability and plasticity of oospore production in the five *Phytophthora infestans* populations sampled along an altitudinal gradient (ranged from lowest altitude at the site A to highest altitude at the site E) of the Dongshan Mountain located in Xuanwei, Yunnan, China.

Sites	Heritability	Plasticity	P/H^a^
A	0.041	0.831	20.46
B	0.022	0.702	32.42
C	0.062	0.746	12.00
D	0.007	0.734	99.11
E	0.021	0.705	33.86
Average	0.029	0.736	25.28

^a^ Ratio of plasticity to heritability.

### 3.4 Population differentiation in SSR and oospore production

Pairwise population differentiation (*F*_ST_) in SSR markers ranged from 0.022 to 0.203 between the pathogen sampled from different altitudes and the pairwise population differentiation (*Q*_ST_) among the pathogen sampled from different altitudes in oospore production ranged from 0.000 to 0.417 (Table 4). The overall *Q*_ST_ was 0.179, which was higher than overall *F*_ST_ (0.147) but the difference between the two population differentiations was not significant by a two‐tailed *t*‐test (*p* < 0.073).

**TABLE 4:** Pairwise comparison between population differentiation in SSR marker loci (*F*_ST_) and oospore production (*Q*_ST_) among *P. infestans* populations sampled from five altitudinal sites (ranging from lowest altitude at site A to highest altitude at site E) along the Dongshan Mountain located in Xuanwei, Yunnan, China.

Population	A	B	C	D	E
A	*	0.146	0.053	0.056	0.203
B	0.314	*	0.084	0.078	0.188
C	0.000	0.297	*	0.022	0.082
D	0.000	0.377	0.000	*	0.086
E	0.359	0.000	0.319	0.417	*

*Note*: Values above the diagonal are *F*_ST_ and values below the diagonal are *Q*_ST_.

## 4 DISCUSSION

As an important environmental parameter, temperature can have crucial impact on nearly all biological processes (Dysthe et al., 2015; Shaffer et al., 2020). In this study, we used a statistical genetic approach to assess the contribution of both contemporary and historical temperatures to oospore production in self‐fertile isolates of *P. infestans*. The contemporary effects, reflecting the physiological adaptation of the pathogen attributable to gene expression and metabolic regulations, were evaluated by comparing oospore production of the pathogen at different experimental temperatures. The historical effects, reflecting genetic adaptation of the pathogen associated with genomic change, were assessed by comparing oospore production of the pathogen sampled along the altitudinal gradient of a mountain. On average, air temperature decreases by 6.5°C with a 1000 m rise in sea level (Smithson et al., 2013) and altitude is considered a good proxy for historical temperatures encountered by a species (Bahram et al., 2012). Statistical analyses reveal that contemporary temperature, historical temperature (altitude), pathogen genetics (isolates) and their interaction all contribute greatly to the thermal adaptation of oospore production (Table S2). Further analysis by variance partitioning shows that genetic variation in the pathogen as measured by heritability (Scheiner & Lyman, 1989) accounts for only 3% of the phenotypic variation in oospore production, while the interaction between pathogen genetics and temperature quantified by plasticity (Scheiner & Lyman, 1989) accounts for ~75% of the phenotypic variation. These results indicate that the main factor determining oospore production of *P. infestans* is contemporary temperature and suggest that thermally regulated gene expression and other bioprocesses such as modifications of post‐translational processes and biochemical pathways are more important than genetic architecture in influencing the reproductive behaviours of the pathogen, consistent with thermal adaptation pattern for functional traits in many species (Maynard et al., 2019; Zhan & McDonald, 2011).

According to common belief in mycology, sexual reproduction of facultative fungi and oomycetes, such as many eukaryotic plant pathogens, is induced by environmental stress during off‐seasons with unfavourable temperature, nutrient conditions, etc. (Chern & Ko, 1994; Cohen et al., 1997). In *P. infestans*, a previous study reported that oospores were mainly formed at temperatures <15°C (Cohen et al., 1997), suggesting that lower temperatures are preferred for the sexual reproduction of the pathogen. As a comparison, the optimum temperatures for the pathogen infection and its host growth were recorded as 16–21°C (Seidl Johnson et al., 2015) and 15–22°C (Maziero et al., 2009) respectively. This ‘cold’ preference feature of oospore production was thought to be an important factor responsible for the unique characteristics of Nordic potato late blight epidemics, which are mainly initiated by oospores as primary inoculum (Andersson et al., 2009).

However, our results disagree with both the theoretical expectation (Turkensteen et al., 2000) and previous empirical observations (Cohen et al., 1997) regarding the thermal biology of oospore production in *P. infestans*. Only a few oospores were detected at the experimental temperatures below 16°C. The quadratic model based on the data generated from the five experimental temperatures predicts that the average optimal temperature of oospore production is ~21°C, which is similar to the optimal temperatures for *P. infestans* infection and potato growth. The model also reveals that oospore production shares thermal profile (spectrum and shape) with potato growth and *P. infestans* infection, but skewing towards higher temperatures (Figure 3, Wu et al., 2022; Yang et al., 2016). Taken together, these results indicate that the temperatures required for *P. infestans* to produce oospores are much higher than reported (Cohen et al., 1997; Turkensteen et al., 2000). They also suggest that the pathogen could reproduce sexually throughout the parasitic stages of their life cycle, contrary to theoretical expectations (Clément et al., 2010) but consistent with field observations. For example, we found that the proportion of infected potato leaves containing oospores increased from ~10% in the early season to ~80% in the late seasons under field conditions in Sweden (BA, unpublished data). Sexual reproduction under favourable environmental conditions in the parasitic stage of pathogens when hosts are not the constraining factor for nutrient has also been reported in other pathogens such as *Zymoseptoria tritici* (Hassine et al., 2019; Zhan et al., 1998) and *Phaeosphaeria nodorum* (Sommerhalder et al., 2011).

Global warming is expected to increase both temperature averages and fluctuations (Allen et al., 2018). We show that the projected maximum oospore production in *P. infestans* is positively associated with the projected optimum temperature and thermal breadth. These results not only further support our hypothesis that ‘coldness’ is not necessarily the trigger for sexual reproduction in *P. infestans* but also suggest that the natural selection driven by the ongoing global warming may increase the representation of sexual offspring in *P. infestans* populations, thereby enhancing their ability to generate new genotypic variation for ecological adaptation (Runno‐Paurson et al., 2022). This should be of particular concern for pathogenic fungi and oomycetes in which the coexistence of two counterpart mating types spatially and temporally is not required for sexual reproduction (Zhu et al., 2016). Despite being classified as a heterothallic pathogen, self‐fertile *P. infestans* has been reported in many countries (Retes‐Manjarrez et al., 2022; Smart et al., 2000) and even dominates in some surveys such as the places we collected samples for this study (Zhu et al., 2016).

Interestingly, although the analysis of variance indicates that adaptation to historical temperature (e.g. altitudinal effect) contributes substantially to oospore production of the pathogen, similar population differentiation was found in oospore production measured by *Qst* and in the SSR markers measured by *F*_ST_. Two events may explain the unexpected observations. First, the constant exchange of genetic material among pathogen populations at different altitudes dilutes the genetic divergence otherwise built up by natural selection. In addition, the higher regions receive primary inoculum from lowlands each year. This upwards gene flow of the pathogen is common in Yunnan (Yang et al., 2022) due to its year‐round potato production model. This type of gene flow has also been documented in other *Phytophthora* species (Jung et al., 2017). However, this interpretation contradicts our recent observations of genetic variation in an effector gene and UV adaptation of the pathogen, showing a clear pattern of altitudinal distribution (Wang et al., 2021; Yang et al., 2022). On the other hand, the observed pattern of altitudinal distribution in oospore production may be generated by the interaction between UV and thermal adaptation. It is widely believed that UV radiation induces sexual reproduction in mycelial pathogen (Palmer et al., 2021). As altitude increases, UV radiation increases but air temperature decreases (Blumthaler et al., 1992). At high altitudes, higher UV radiation induces oospore production of the pathogen but lower temperatures reduce the production. The interactive effect between altitudes and temperatures may also explain the differential response of altitude isolates to experimental temperature and the more important role of altitude–temperature than isolate–temperature interaction in regulating oospore production.

## 5 CONCLUSION

We find both historical and contemporary temperatures contribute to sexual reproduction of *P. infestans*. We also find that *P. infestans* requires higher temperatures than previous reports for sexual reproduction and could occur throughout potato production seasons due to the shared thermal profiles among oospore production, pathogen infection and potato growth. However, whether or not sexual reproduction will translate into epidemic risk depends on the germination and vitality of oospores produced and the trade‐offs between the gains/losses for pathogenicity and other ecological adaptations (Mariette et al., 2016). Furthermore, the current result is derived from homothallic *P. infestans*. Whether the oospore production pattern is also present in its heterothallic counterpart needs to be confirmed.

## CONFLICT OF INTEREST STATEMENT

The authors have no conflict of interest to declare.

## Supporting information


Tables S1–S3.

## Data Availability

All data generated in this study are included in the Supplementary Files.
