# Supplementary material for: Evaluating the contribution of historical and contemporary temperature to the oospore production of self‐fertile Phytophthora infestans
Source: Evol Appl. 2024 Jan 29;17(1):e13643. doi: 10.1111/eva.13643 (PMC10824702; doi:10.1111/eva.13643)
Supplement: Supplementary file 1 — Tables S1–S3. [file EVA-17-e13643-s001.docx]

**Table S1** Number of oospores detected in the 116 isolates sampled from five altitudes along Dongshan Mountain located in Xuanwei, Yunnan, China, and tested at five experimental temperatures (Note: the first letter in the isolate name represents the altitude/population from which the isolate was sampled, i.e., A from 1975 m, B from 2124 m, C from 2471 m, D from 2591 m and E from 2677 m).

| Isolates | 13℃ | | | 16℃ | | | 19℃ | | | 22℃ | | | 25℃ | | |
| --- | --- | --- | --- | --- | --- | --- | --- | --- | --- | --- | --- | --- | --- | --- | --- |
|  | Rep 1 | Rep 2 | Rep 3 | Rep 1 | Rep 2 | Rep 3 | Rep 1 | Rep 2 | Rep 3 | Rep 1 | Rep 2 | Rep 3 | Rep 1 | Rep 2 | Rep 3 |
| ADS3-10 | 4 | 1 | 1 | 63 | 47 | 61 | 465 | 316 | 340 | 99 | 114 | 98 | 1 | 0 | 12 |
| ADS3-5 | 0 | 0 | 0 | 15 | 24 | 37 | 19 | 21 | 25 | 51 | 34 | 41 | 0 | 0 | 0 |
| ADS3-6 | 0 | 0 | 0 | 1 | 1 | 7 | 9 | 10 | 9 | 42 | 83 | 51 | 0 | 0 | 0 |
| ADS3-7 | 0 | 0 | 0 | 66 | 32 | 29 | 17 | 18 | 12 | 158 | 133 | 122 | 0 | 0 | 0 |
| ADS3-9 | 3 | 3 | 4 | 31 | 42 | 52 | 47 | 52 | 53 | 481 | 680 | 504 | 0 | 0 | 0 |
| AH8-1 | 0 | 0 | 0 | 0 | 0 | 0 | 6 | 3 | 4 | 62 | 40 | 28 | 0 | 0 | 0 |
| AH8-6 | 0 | 1 | 0 | 9 | 5 | 2 | 19 | 19 | 17 | 328 | 208 | 132 | 0 | 0 | 0 |
| AH9-2 | 0 | 0 | 0 | 1 | 0 | 2 | 8 | 6 | 5 | 28 | 33 | 24 | 0 | 0 | 0 |
| AH9-4 | 2 | 2 | 1 | 6 | 17 | 27 | 208 | 181 | 92 | 296 | 272 | 389 | 0 | 2 | 0 |
| AL-2 | 0 | 2 | 2 | 6 | 7 | 6 | 30 | 28 | 27 | 69 | 55 | 35 | 0 | 0 | 0 |
| AL-3 | 0 | 0 | 0 | 2 | 1 | 4 | 14 | 16 | 20 | 15 | 16 | 8 | 2 | 2 | 0 |
| AL-6 | 0 | 0 | 0 | 30 | 12 | 41 | 4 | 5 | 5 | 420 | 260 | 248 | 0 | 1 | 0 |
| AXS4-1 | 0 | 0 | 0 | 126 | 83 | 47 | 16 | 14 | 23 | 0 | 2 | 1 | 0 | 0 | 0 |
| AXS4-10 | 1 | 0 | 0 | 0 | 1 | 1 | 12 | 12 | 18 | 136 | 128 | 152 | 0 | 0 | 0 |
| AXS4-2 | 0 | 1 | 0 | 51 | 5 | 16 | 23 | 17 | 170 | 520 | 402 | 344 | 1 | 0 | 1 |
| AXS4-3 | 0 | 0 | 0 | 3 | 1 | 2 | 35 | 18 | 19 | 204 | 244 | 268 | 0 | 0 | 0 |
| AXS5-3 | 0 | 0 | 0 | 3 | 2 | 10 | 55 | 34 | 56 | 448 | 340 | 520 | 0 | 0 | 0 |
| AXS5-5 | 0 | 0 | 0 | 14 | 4 | 6 | 9 | 12 | 18 | 260 | 340 | 284 | 0 | 0 | 0 |
| AYS1-3 | 0 | 0 | 0 | 0 | 0 | 0 | 1 | 0 | 2 | 33 | 32 | 25 | 0 | 0 | 0 |
| AYS1-4 | 0 | 0 | 0 | 2 | 1 | 7 | 2 | 5 | 5 | 18 | 39 | 46 | 0 | 0 | 0 |
| AYS1-5 | 0 | 0 | 0 | 10 | 5 | 9 | 105 | 103 | 112 | 250 | 320 | 260 | 0 | 0 | 0 |
| AYS4-10 | 1 | 0 | 1 | 7 | 6 | 5 | 7 | 11 | 8 | 17 | 9 | 5 | 0 | 0 | 2 |
| AYS4-11 | 1 | 0 | 0 | 2 | 6 | 0 | 51 | 58 | 62 | 9 | 5 | 6 | 0 | 0 | 0 |
| AYS5-2 | 0 | 0 | 0 | 34 | 23 | 8 | 17 | 17 | 15 | 420 | 220 | 570 | 0 | 0 | 0 |
| AYS5-7 | 1 | 0 | 1 | 0 | 1 | 1 | 15 | 13 | 12 | 28 | 168 | 172 | 0 | 0 | 0 |
| B-HYW-12 | 7 | 17 | 6 | 18 | 63 | 32 | 158 | 169 | 211 | 568 | 528 | 424 | 0 | 1 | 0 |
| B-HYW-13 | 0 | 0 | 0 | 4 | 5 | 3 | 278 | 210 | 248 | 64 | 56 | 58 | 0 | 0 | 0 |
| B-HYW-14 | 3 | 0 | 0 | 8 | 13 | 5 | 2 | 1 | 3 | 232 | 148 | 216 | 0 | 0 | 0 |
| B-HYW-15 | 0 | 0 | 0 | 7 | 5 | 7 | 0 | 2 | 2 | 124 | 88 | 80 | 0 | 0 | 0 |
| B-HYW-18 | 0 | 0 | 0 | 1 | 1 | 0 | 1 | 2 | 5 | 268 | 132 | 196 | 0 | 0 | 0 |
| B-HYW-2 | 2 | 0 | 0 | 1 | 0 | 2 | 3 | 7 | 12 | 704 | 416 | 448 | 0 | 0 | 0 |
| B-HYW-21 | 0 | 0 | 0 | 15 | 24 | 13 | 14 | 18 | 39 | 132 | 252 | 188 | 0 | 1 | 0 |
| B-HYW-30 | 0 | 0 | 0 | 9 | 11 | 6 | 13 | 20 | 132 | 784 | 545 | 665 | 0 | 2 | 1 |
| B-HYW-5 | 0 | 0 | 0 | 6 | 16 | 7 | 27 | 45 | 37 | 515 | 530 | 415 | 1 | 1 | 2 |
| B-HYW-6 | 0 | 0 | 0 | 36 | 16 | 14 | 25 | 22 | 23 | 38 | 27 | 64 | 0 | 0 | 0 |
| B-HYW-7 | 0 | 0 | 0 | 0 | 2 | 1 | 0 | 3 | 3 | 21 | 22 | 18 | 0 | 0 | 0 |
| B-HYW-8 | 0 | 1 | 0 | 5 | 4 | 2 | 11 | 6 | 7 | 9 | 5 | 10 | - | 0 | 0 |
| B-HYW-9 | 0 | 1 | 1 | 8 | 4 | 8 | 1 | 2 | 0 | 260 | 324 | 780 | 0 | 0 | 1 |
| B-KY-1 | 8 | 4 | 12 | 15 | 20 | 14 | 71 | 69 | 98 | 468 | 488 | 412 | 0 | 1 | 1 |
| B-KY-18 | 1 | 0 | 1 | 4 | 2 | 8 | 6 | 12 | 12 | 240 | 252 | 352 | 0 | 0 | 0 |
| B-KY-2 | 0 | 0 | 0 | 84 | 81 | 5 | 11 | 12 | 18 | 448 | 441 | 600 | 0 | 0 | 0 |
| B-KY-3 | 0 | 0 | 0 | 2 | 0 | 4 | 0 | 1 | 2 | 320 | 421 | 262 | 0 | 0 | 0 |
| B-KY-5 | 0 | 0 | 2 | 5 | 4 | 6 | 0 | 0 | 0 | 102 | 368 | 72 | 1 | 0 | 0 |
| B-XS2-10 | 0 | 0 | 1 | 12 | 5 | 5 | 14 | 15 | 45 | 116 | 84 | 104 | 0 | 0 | 0 |
| B-XS2-11 | 0 | 0 | 0 | 34 | 42 | 16 | 54 | 37 | 50 | 768 | 576 | 162 | 0 | 0 | 0 |
| B-XS2-12 | 0 | 0 | 0 | 7 | 7 | 12 | 10 | 14 | 11 | 504 | 345 | 320 | 0 | 1 | 0 |
| B-XS2-14 | 0 | 0 | 0 | 5 | 14 | 2 | 0 | 2 | 1 | 56 | 28 | 19 | 0 | 0 | 0 |
| B-XS2-17 | 3 | 0 | 0 | 16 | 12 | 112 | 59 | 44 | 47 | 48 | 42 | 24 | 0 | 0 | 0 |
| B-XS2-18 | 0 | 0 | 0 | 29 | 33 | 25 | 19 | 7 | 12 | 25 | 108 | 67 | 0 | 0 | 0 |
| B-XS2-19 | 0 | 0 | 0 | 14 | 17 | 12 | 46 | 34 | 41 | 848 | 736 | 288 | 0 | 0 | 0 |
| B-XS2-2 | 0 | 0 | 0 | 0 | 0 | 0 | 3 | 0 | 3 | 56 | 63 | 22 | 2 | 0 | 0 |
| B-XS2-9 | 1 | 0 | 0 | 2 | 7 | 5 | 1 | 0 | 5 | 384 | 324 | 19 | 0 | 0 | 2 |
| CHZ-1 | 0 | 0 | 0 | 0 | 0 | 0 | 14 | 10 | 16 | 184 | 168 | 112 | 0 | 0 | 0 |
| CHZ-11 | 0 | 0 | 0 | 0 | 0 | 0 | 2 | 1 | 0 | 0 | 3 | 0 | 0 | 0 | 0 |
| CHZ-12 | 3 | 1 | 0 | 31 | 48 | 0 | 193 | 141 | 383 | 576 | 560 | 272 | 0 | 0 | 5 |
| CHZ-13 | 0 | 0 | 0 | 14 | 6 | 28 | 47 | 56 | 77 | 480 | 368 | 448 | 0 | 1 | 0 |
| CHZ-15 | 0 | 0 | 0 | 0 | 0 | 0 | 6 | 22 | 27 | 13 | 3 | 5 | 0 | 0 | 0 |
| CHZ-17 | 0 | 0 | 0 | 0 | 0 | 0 | 0 | 0 | 0 | 0 | 1 | 1 | 0 | 0 | 0 |
| CHZ-18 | 3 | 2 | 5 | 3 | 1 | 0 | 126 | 77 | 72 | 312 | 324 | 42 | 0 | 0 | 0 |
| CHZ-27 | 1 | 0 | 0 | 10 | 6 | 4 | 38 | 36 | 45 | 13 | 23 | 129 | 0 | 0 | 0 |
| CHZ-34 | 0 | 0 | 0 | 0 | 0 | 0 | 1 | 0 | 1 | 1 | 0 | 1 | 0 | 0 | 0 |
| CHZ-35 | 0 | 0 | 0 | 7 | 3 | 2 | 42 | 54 | 33 | 0 | 3 | 1 | 0 | 0 | 0 |
| CHZ-36 | 0 | 0 | 0 | 1 | 0 | 1 | 6 | 12 | 5 | 20 | 29 | 37 | 0 | 0 | 0 |
| CHZ-37 | 0 | 0 | 0 | 1 | 0 | 0 | 2 | 0 | 0 | 28 | 31 | 17 | 0 | 0 | 0 |
| CHZ-4 | 0 | 0 | 0 | 3 | 0 | 5 | 7 | 4 | 6 | 43 | 29 | 57 | 0 | 0 | 0 |
| CHZ-43 | 6 | 6 | 7 | 9 | 12 | 11 | 20 | 27 | 18 | 784 | 464 | 240 | 0 | 0 | 0 |
| CHZ-44 | 0 | 0 | 0 | 0 | 0 | 0 | 3 | 8 | 4 | 0 | 0 | 0 | - | 0 | 0 |
| CHZ-45 | 0 | 0 | 0 | 2 | 4 | 4 | 20 | 50 | 30 | 464 | 800 | 736 | 0 | 0 | 0 |
| CHZ-55 | 1 | 0 | 0 | 5 | 3 | 3 | 4 | 2 | 5 | 21 | 2 | 3 | 0 | 0 | 0 |
| CHZ-56 | 0 | 0 | 0 | 2 | 1 | 3 | 40 | 22 | 25 | 55 | 54 | 91 | 0 | 0 | 0 |
| CHZ-59 | 0 | 0 | 0 | 1 | 2 | 3 | 1 | 1 | 3 | 8 | 5 | 7 | 0 | 0 | 0 |
| CHZ-61 | 0 | 0 | 0 | 1 | 1 | 2 | 32 | 16 | 48 | 124 | 192 | 402 | 0 | 0 | 0 |
| CHZ-62 | 0 | 0 | 1 | 1 | 7 | 3 | 7 | 13 | 13 | 300 | 260 | 192 | 0 | 0 | 0 |
| CHZ-66 | 1 | 0 | 1 | 26 | 48 | 17 | 76 | 96 | 93 | 0 | 0 | 0 | 0 | 0 | 0 |
| CHZ-70 | 0 | 0 | 0 | 9 | 15 | 9 | 96 | 115 | 87 | 512 | 320 | 528 | 0 | 0 | 0 |
| CHZ-8 | 4 | 12 | 9 | 27 | 8 | 14 | 73 | 86 | 57 | 184 | 186 | 264 | 0 | 0 | 0 |
| CHZ-9 | 0 | 0 | 0 | 7 | 5 | 13 | 129 | 132 | 141 | 49 | 128 | 144 | 0 | 0 | 0 |
| DQS9-14 | 0 | 0 | 0 | 0 | 1 | 2 | 6 | 4 | 2 | 96 | 80 | 144 | 0 | 0 | 0 |
| DQS9-17 | 0 | 0 | 1 | 1 | 2 | 5 | 14 | 3 | 6 | 140 | 272 | 196 | 1 | 0 | 1 |
| DQS9-18 | 0 | 0 | 0 | 0 | 0 | 1 | 13 | 10 | 8 | 60 | 28 | 41 | 0 | 0 | 0 |
| DQS9-20 | 0 | 1 | 0 | 0 | 3 | 1 | 8 | 22 | 10 | 82 | 36 | 48 | 0 | 0 | 0 |
| DQS9-5 | 0 | 0 | 2 | 1 | 2 | 0 | 18 | 16 | 46 | 68 | 44 | 52 | 0 | 0 | 0 |
| DQS9-8 | 0 | 3 | 1 | 7 | 10 | 6 | 16 | 67 | 35 | 80 | 48 | 28 | 0 | 1 | 0 |
| DXS2-12 | 0 | 0 | 0 | 3 | 2 | 1 | 18 | 18 | 18 | 168 | 240 | 204 | 0 | 0 | 0 |
| DXS2-16 | 0 | 0 | 0 | 0 | 0 | 0 | 7 | 6 | 9 | 7 | 8 | 5 | 0 | 0 | 0 |
| DXS2-17 | 0 | 0 | 0 | 15 | 5 | 6 | 13 | 12 | 8 | 128 | 96 | 128 | 3 | 1 | 0 |
| DXS2-23 | 1 | 0 | 0 | 3 | 5 | 1 | 61 | 96 | 65 | 96 | 76 | 104 | 0 | 0 | 0 |
| DXS2-24 | 0 | 0 | 0 | 1 | 2 | 6 | 6 | 7 | 3 | 864 | 120 | 976 | 0 | 0 | 0 |
| DXS2-31 | 0 | 0 | 0 | 0 | 0 | 0 | 2 | 4 | 1 | 2 | 4 | 2 | 0 | 1 | 1 |
| DXS2-33 | 0 | 0 | 0 | 3 | 4 | 7 | 28 | 312 | 241 | 88 | 64 | 68 | 2 | 0 | 1 |
| DXS2-41 | 1 | 0 | 2 | 4 | 7 | 5 | 101 | 103 | 120 | 476 | 453 | 177 | 0 | 0 | 0 |
| DXS2-43 | 0 | 0 | 0 | 22 | 7 | 41 | 46 | 52 | 35 | 752 | 736 | 784 | 1 | 0 | 2 |
| DXS2-8 | 0 | 0 | 0 | 9 | 12 | 17 | 84 | 68 | 109 | 11 | 15 | 80 | 0 | 0 | 0 |
| DXS2-9 | 1 | 1 | 1 | 7 | 9 | 3 | 13 | 13 | 10 | 3 | 1 | 2 | 0 | 0 | 0 |
| EHZ-1 | 0 | 0 | 0 | 1 | 1 | 0 | 3 | 9 | 8 | 272 | 512 | 336 | 0 | 0 | 1 |
| EHZ-3 | 0 | 1 | 0 | 1 | 0 | 0 | 5 | 8 | 13 | 560 | 504 | 765 | 0 | 0 | 0 |
| EHZ-7 | 3 | 2 | 2 | 1 | 8 | 6 | 4 | 2 | 18 | 224 | 272 | 172 | 0 | 0 | 0 |
| EHZ-8 | 0 | 0 | 1 | 2 | 4 | 1 | 101 | 98 | 83 | 656 | 624 | 240 | 0 | 0 | 0 |
| EHZ-9 | 0 | 0 | 0 | 2 | 1 | 1 | 9 | 12 | 20 | 2 | 4 | 5 | 0 | - | 0 |
| EYS5-2 | 0 | 0 | 0 | 1 | 1 | 0 | 47 | 55 | 43 | 224 | 124 | 292 | 0 | 0 | 0 |
| EYS9-1 | 2 | 0 | 2 | 2 | 2 | 6 | 11 | 22 | 11 | 576 | 625 | 256 | 1 | 0 | 2 |
| EYS9-10 | 0 | 1 | 0 | 0 | 2 | 0 | 0 | 1 | 1 | 108 | 152 | 121 | 0 | 0 | 0 |
| EYS9-12 | 0 | 0 | 0 | 0 | 3 | 1 | 1 | 2 | 3 | 48 | 172 | 112 | 0 | 0 | 0 |
| EYS9-13 | 0 | 2 | 0 | 4 | 1 | 3 | 64 | 63 | 213 | 192 | 124 | 101 | 0 | 0 | 3 |
| EYS9-14 | 0 | 0 | 0 | 1 | 1 | 0 | 56 | 24 | 27 | 10 | 11 | 24 | 0 | - | 0 |
| EYS9-16 | 0 | 0 | 0 | 3 | 3 | 4 | 198 | 186 | 176 | 104 | 152 | 172 | 0 | 0 | 0 |
| EYS9-18 | 0 | 0 | 0 | 5 | 3 | 2 | 70 | 69 | 335 | 816 | 720 | 864 | 0 | 0 | 1 |
| EYS5-1 | 0 | 0 | 0 | 2 | 2 | 2 | 29 | 29 | 46 | 706 | 156 | 864 | 0 | 0 | 3 |
| EYS9-28 | 0 | 0 | 0 | 1 | 3 | 7 | 30 | 24 | 40 | 480 | 72 | 272 | 2 | 0 | 0 |
| EYS9-3 | 1 | 0 | 1 | 9 | 16 | 8 | 84 | 102 | 95 | 98 | 56 | 44 | 1 | 0 | 0 |
| EYS9-39 | 0 | 0 | 0 | 0 | 0 | 1 | 0 | 1 | 1 | 112 | 88 | 92 | 0 | 0 | 0 |
| EYS9-4 | 0 | 0 | 0 | 1 | 0 | 0 | 11 | 18 | 7 | 336 | 528 | 480 | 0 | 0 | 0 |
| EYS9-41 | 0 | 0 | 1 | 3 | 7 | 4 | 4 | 6 | 0 | 312 | 352 | 112 | 0 | 0 | 0 |
| EYS9-43 | 0 | 0 | 0 | 0 | 0 | 2 | 2 | 1 | 5 | 39 | 66 | 21 | 0 | 0 | 0 |
| EYS9-53 | 0 | 0 | 0 | 2 | 2 | 0 | 5 | 10 | 3 | 60 | 44 | 152 | 0 | 0 | 1 |
| EYS9-7 | 0 | 0 | 0 | 2 | 3 | 9 | 49 | 50 | 68 | 624 | 496 | 480 | 0 | 0 | 0 |

“ –“ represents missing data due to contamination.

**Table S2** Analysis of variance (ANOVA) for oospore production measured on the 15^th^ day after inoculation at five temperature regimes in the *Phytophthora infestans* populations sampled from five different altitudinal locations along Dongshan mountain in Xuanwei, Yunnan.

|  | **Source** | **D.F** | **F value** | **P** |
| --- | --- | --- | --- | --- |
|  | **Altitude** | 4 | 18.58 | < .0001 |
|  | **Temperature** | 4 | 1121.93 | < .0001 |
|  | **Isolate** | 111 | 12.30 | < .0001 |
|  | **Altitude x Temperature** | 16 | 20.31 | < .0001 |
|  | **Isolate x Temperature** | 444 | 10.40 | < .0001 |
|  | **Error** | 1160 |  |  |

**Table S3** Analysis of variance (ANOVA) for the thermal biology of oospore production in the *Phytophthora infestans* populations sampled from five altitudinal sites along Dongshan mountain located in Xuanwei, Yunnan, China.

| **Source** | **DF** | **SS** | **Mean SS** | **F-Value** | **P-value** |
| --- | --- | --- | --- | --- | --- |
| **OT_opt_** |  |  |  |  |  |
| Altitude | 4 | 75.61 | 18.90 | 5.02 | 0.0007 |
| Isolate | 111 | 516.60 | 4.65 | 1.24 | 0.0919 |
| Error | 232 |  |  |  |  |
| **OT_max_** |  |  |  |  |  |
| Altitude | 4 | 157.10 | 39.27 | 5.50 | 0.0003 |
| Isolate | 111 | 1013.19 | 9.13 | 1.28 | 0.0616 |
| Error | 232 |  |  |  |  |
| **OT_min_** |  |  |  |  |  |
| Altitude | 4 | 25.50 | 6.38 | 3.73 | 0.0058 |
| Isolate | 111 | 213.98 | 1.93 | 1.13 | 0.2245 |
| Error | 232 |  |  |  |  |
| **OT_b_** |  |  |  |  |  |
| Altitude | 4 | 62.75 | 15.69 | 5.95 | 0.0001 |
| Isolate | 111 | 387.95 | 3.50 | 1.33 | 0.0382 |
| Error | 232 |  |  |  |  |
